# The Continuous Assessment of Cranial Motion in Thermoplastic Masks During CyberKnife Radiosurgery for Trigeminal Neuralgia

**DOI:** 10.7759/cureus.607

**Published:** 2016-05-12

**Authors:** Tewfik J Bichay, Alan Mayville

**Affiliations:** 1 The Lacks Cancer Center, Radiation Oncology, Mercy Health, Saint Mary's, Grand Rapids, Michigan, USA

**Keywords:** radiosurgery, srs, trigeminal neuralgia, stereotactic radiosurgery, thermoplastic mask, treatment motion

## Abstract

Stereotactic radiosurgery (SRS) treatment is characterized by high doses per fraction and extremely steep dose gradients. This requires a great degree of accurate localization to the appropriate treatment position, and continuous immobilization during the treatment session. In the case of Trigeminal Neuralgia (TGN) treatment this is especially true as the very small target volume makes positional accuracy critical. In this study we carried out a quantitative analysis of patient motion during the full treatment fraction within a radiosurgery immobilization mask system. Patient cranial movement was assessed by using the image guidance stereo x-ray cameras on a CyberKnife (CK) M6 robotic radiosurgery system (Accuray, Sunnyvale, CA). A total of five patients received treatments for either right or left TGN. The duration of treatment varied from 24-64 minutes. Orthogonal images were taken every 15 seconds during the treatment to assess patient movement. Approximately 60 stereo images were taken per patient and a total of 560 images were analyzed in this study. The mean absolute movement in each of longitudinal, lateral or vertical directions was approximately 0.3 mm for the duration of the treatment; however, on occasion much greater movement was observed during a fraction. The maximum displacement was in the longitudinal direction and reached 2.4 mm compared to the initial setup. Images taken at the end of the treatment session showed that the patients typically return to a position closer to the original setup position than the maximum excursion that occurred. This data suggests that although this mask system appears stable during much of the treatment session; for some patients there may be momentary patient movements that take place. Frequent imaging and correction can help mitigate the effect of this movement. It is important to understand the limitations of non-invasive mask systems when used for very high precision treatment.

## Introduction

Intracranial radiosurgery requires delivering high radiation dose to a very specific localized area while at the same time keeping the patient immobilized. Historically localization and immobilization were achieved with invasive devices such as the halo and Brown-Roberts-Wells (BRW) frames. These frames can accurately localize the cranium to within 1 mm of the target area [1,2]. Unfortunately, these devices are also highly invasive since they require that screws be inserted into the patient’s skull. This invasive nature makes it difficult to fractionate therapy, putting the patient at an inherent disadvantage when receiving therapy for conditions that are more responsive to multiple treatments. More recently the process of localization and immobilization have been separated into individual functions; localization using image guided radiation therapy (IGRT) and immobilization using a semi-rigid device [2-4]. One of the most common alternatives to traditional invasive immobilizers is a plastic radiosurgery mask that is custom molded for each patient, allowing the device to conform to the patient’s head and face. The mask system does not interfere with x-ray-based IGRT and offers a non-invasive method of immobilization during treatment. A potential drawback to this technology is the risk for greater head movement than would be possible with an invasive frame. While some have argued that submillimeter SRS is not feasible with current technology [5] others have demonstrated that thermoplastic masks can maintain stability within 1 mm in any linear direction [2-4,6]. These studies typically rely on x-ray imaging of the patient at the start of the treatment session with a repeat imaging session at the end of the treatment fraction, or may rely on repeated phantom imaging where no motion would be expected [7]. These approaches may not resolve additional motion that may take place in a human during the full course of the treatment fraction. In this study we determined whether continuous monitoring of patient position, at 15-sec intervals throughout the treatment fraction, would demonstrate any additional motion that would not be apparent by only imaging pre- and post-treatment. We continuously monitored cranial motion during CyberKnife radiosurgery for patients undergoing treatment for trigeminal neuralgia. The intent was to determine the effectiveness of this mask-based system in immobilizing the cranium for the duration of critical radiosurgery.

## Materials and methods

### Patient positioning and immobilization

**Figure 1:**
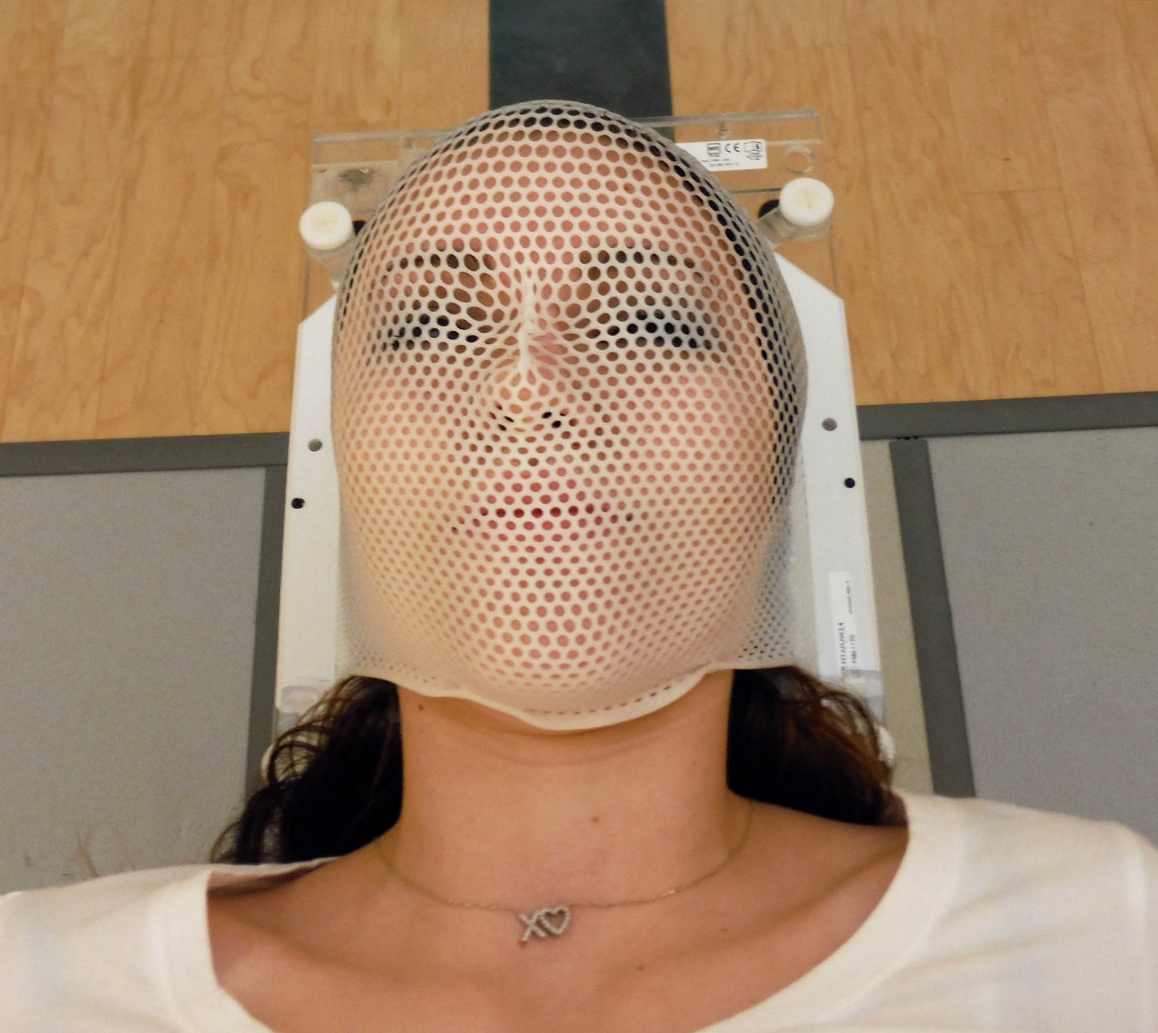
Typical patient setup in thermoplastic mask

All patients were simulated based on our standard CyberKnife cranial protocol. With the patient in a comfortable position, a custom-formed pillow was shaped around the base of the head (Civco Medical, Orange City, Iowa). The lightweight pillow is flexible initially but after water activation becomes solid, holding its shape indefinitely. A semi-rigid 2 mm (Civco) thermoplastic mask was formed over the face and affixed to a plastic frame that is screwed directly to the CK couch for treatment (Figure 1).

### CT/MRI imaging and treatment planning

Computed tomography (CT) Scans were acquired on a Siemens 40-slice Open Sensation using 0.6 mm collimation and image reconstruction of 1 mm steps with a matrix of 512 x 512. The typical scan field-of-view used was a 400 mm x 400 mm resulting in a voxel resolution of 0.8 mm x 0.8 mm x 1.0 mm. For delineation of the trigeminal nerve the CT datasets were registered to high resolution MRI T1 and T2 images with reconstruction of 0.8 mm-1 mm. Critical normal structures such as the lens, orbit, optical nerves, chiasm, brainstem and whole brain were identified and contoured. A 1 mm margin (PRV) was added to all normal structures. The trigeminal nerve was identified on the MRI images as it exited the pons and was circumferentially contoured 4 mm off the pons in the nerve root entry zone. A 1 mm margin was placed on the target to determine the planning treatment volume (PTV). Treatment plans were designed targeting a 3 mm length of the nerve as well as the PTV on a Multiplan V 5.1 (Accuray) treatment planning station (TPS). The 5 mm fixed collimator option was used for all treatments. The treatment time, number of beams and total monitor units used are summarized in Table 1. Mulitplan generates multiple series of DRRs from the CT data and uses these to determine patient alignment during treatment.

**Table 1:** Summary of treatment parameters

	Treatment Time (min)	Number of Beams	Total MU
Patient 1	24	39	8,412
Patient 2	64	122	32,887
Patient 3	49	117	20,153
Patient 4	51	98	22,844
Patient 5	40	67	12,022

### CyberKnife patient setup and treatment delivery

Patients were set up on the treatment couch utilizing the custom-formed hardware at the time of simulation. In-room lasers define the center of the imaging system and provide the therapists with a first estimate for patient alignment. Orthogonal kV x-ray pairs were then taken and the images compared to the planning system-generated DRR images. Adjustments were made to x-ray energy, mA, and pulse time to improve image quality. The patient was then manually aligned to within a few millimeters of the final treatment location. The system recommends shifts and rotations including  pitch, roll and yaw which are confirmed and initiated by the therapists. This process continues iteratively until the residual offsets are within acceptable values. For treatment tracking the *6D Skull Tacking* mode was used. The treatment location system (TLS) compares sets of orthogonal x-rays to a series of TPS-generated DRRs. This process identifies the necessary translational shifts and rotations needed to match the patient's position to that defined at the time of simulation. We have previously shown that the precision of the CK imaging system is approximately 0.30 mm (Abstract presented at the World Congress on Medical Physics and Biomedical Engineering June 7-12. IUPESM p 566).  The imaging system of CK allows for an x-ray image and confirmation of the patients position at a defined interval. For this study a frequency of 15 sec was used for all patients. Any residual offset between the patient’s simulation position and that at the time of treatment is accounted for through robotic positional adjustments. At each beam position, the robot adjusts the target location according to the patient offsets for all linear and rotational parameters.

### Data collection and analysis

The resulting translational offsets of each orthogonal x-ray pair taken at 15 sec intervals was stored for each treatment session. This provides the ability to visualize the patient’s motion throughout the treatment. Of interest for this study is the maximum excursion in any direction, from the initial beam-on position, the progressive changes in translational offsets, and the magnitude of patient motion in any direction from one image to the next. This provides a continuous picture, at 15 sec intervals, of patient motion for the duration of the treatment. The vector displacement between two points was calculated by the formula:

$$\nu =\sqrt{\left ( x_{0}-x_{1}\right )^{2}+\left ( y_{0}-y_{1}\right )^{2}+\left ( z_{0}-z_{1}\right )^{2}}$$

Where $\nu$ - is the vector distance

            $x_{0}-x_{1}$ is the shift in the longitudinal direction

            $y_{0}-y_{1}$ is the shift in the lateral direction

            $z_{0}-z_{1}$ is the shift in the vertical direction

## Results

**Figure 2:**
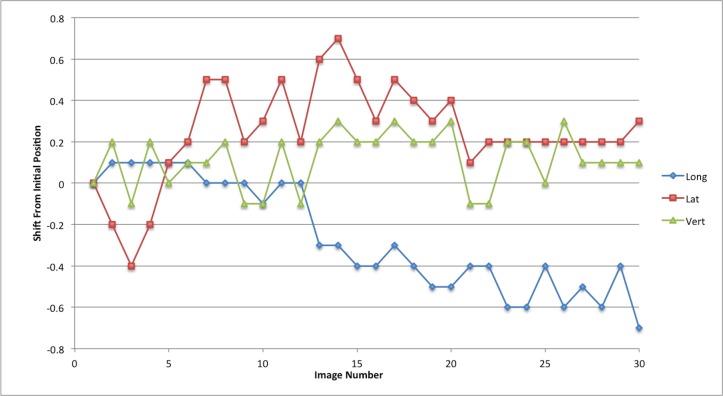
Patient 1 Patient movement over the entire treatment session in longitudinal, lateral and vertical directions. Each point represents the measure of the displacement of the current position compared to the initial setup point

**Figure 3:**
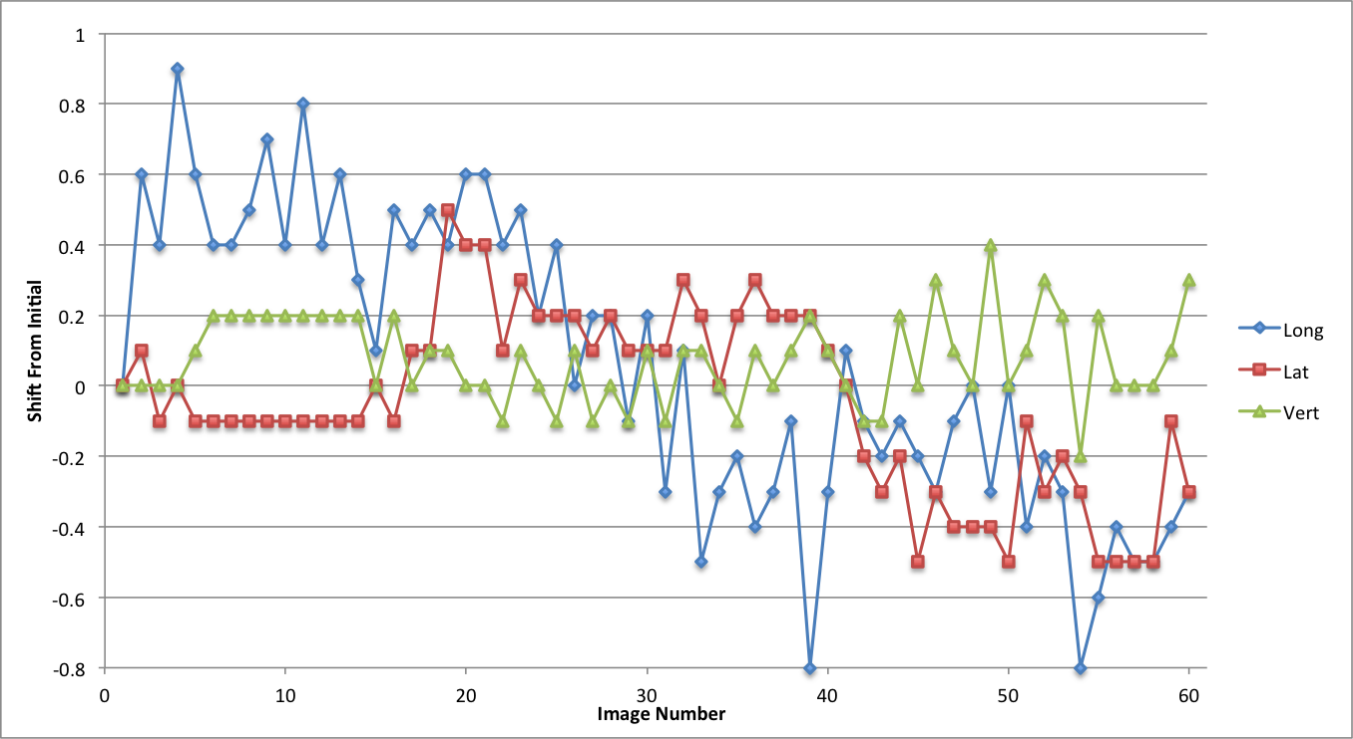
Patient 2 Patient movement over the entire treatment session in longitudinal, lateral and vertical directions. Each point represents the measure of the displacement of the current position compared to the initial setup point

**Figure 4:**
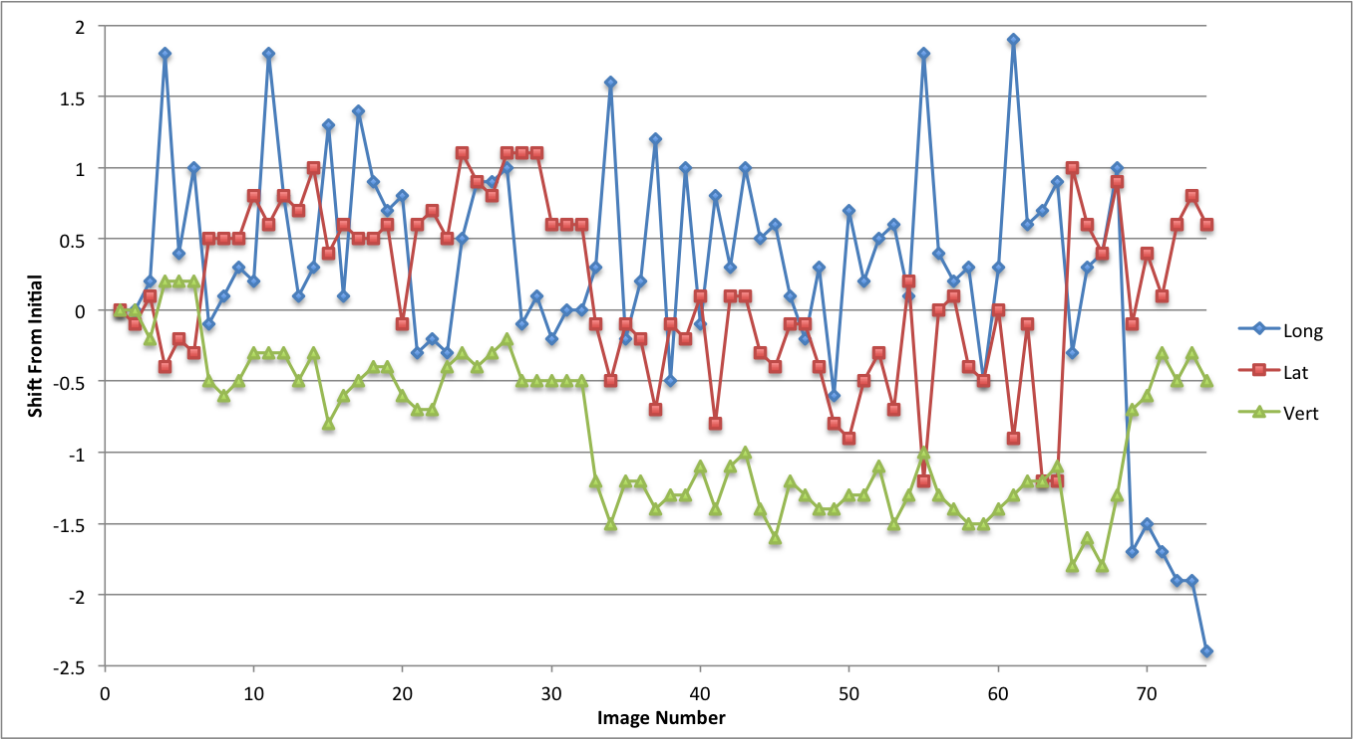
Patient 3 Patient movement over the entire treatment session in longitudinal, lateral and vertical directions. Each point represents the measure of the displacement of the current position compared to the initial setup point

**Figure 5:**
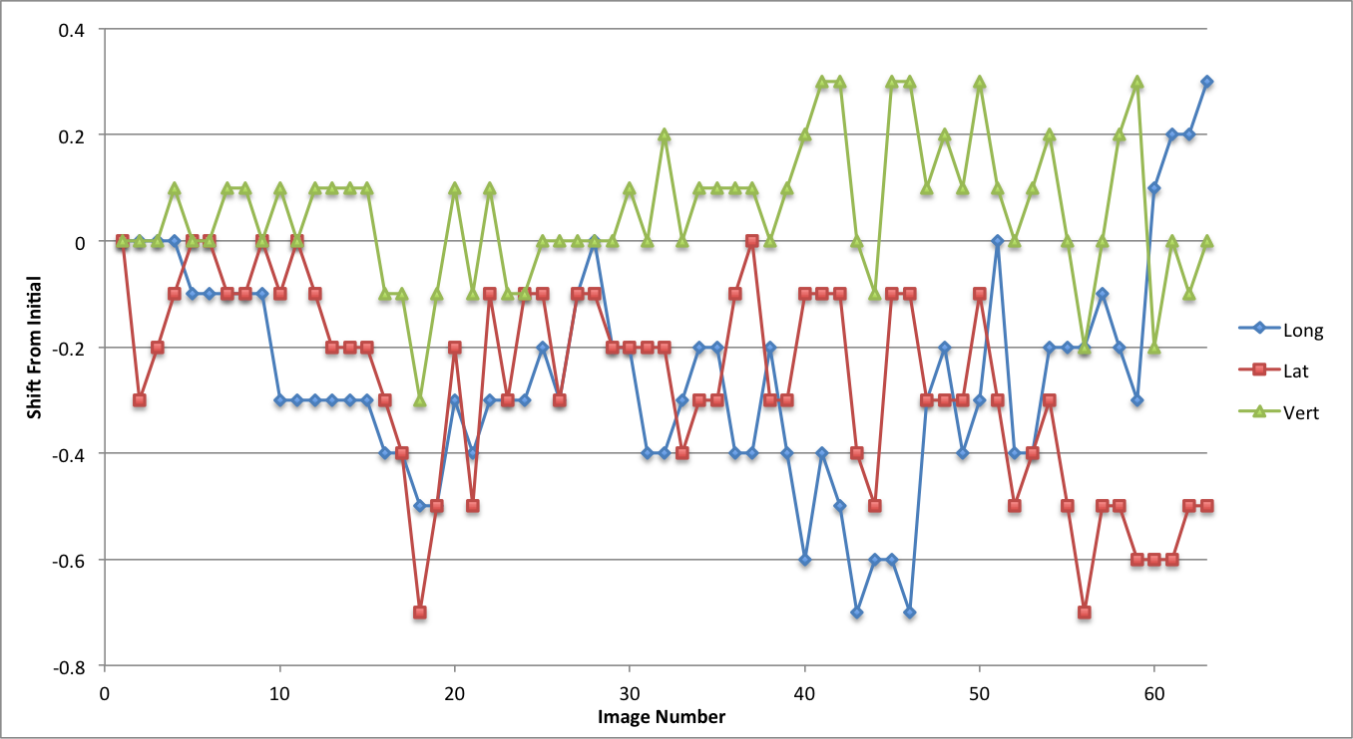
Patient 4 Patient movement over the entire treatment session in longitudinal, lateral and vertical directions. Each point represents the measure of the displacement of the current position compared to the initial setup point

**Figure 6:**
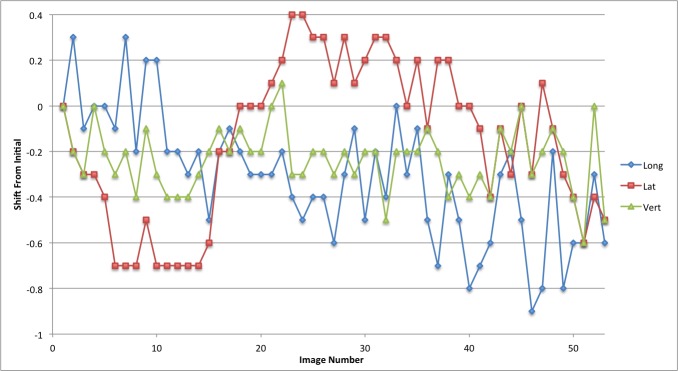
Patient 5 Patient movement over the entire treatment session in longitudinal, lateral and vertical directions. Each point represents the measure of the displacement of the current position compared to the initial setup point

The linear patient motion for the duration of the treatment is presented in graphical form in Figures 2-6. Rotational motion will be discussed in a separate publication. A common approach to motion assessment is to image prior to treatment, with a repeat analysis of position at the completion of the session, and the difference is an indicator of stability. We have analyzed this data in a similar manner, evaluating the final patient position compared to the initial position.

The displacement in this study for pre- and post-session imaging would suggest that movement is typically submillimeter for the majority of patients. With the exception of patient 3, if imaging was carried out only before the start of treatment then again at the completion of the fraction the linear change in any direction would be from 0 mm to 0.7 mm (Table 2; patients 1,2,4 and 5). Since we imaged continuously we could also calculate the maximum error in position during the full session compared to the original starting point.

**Table 2:** Patient displacement relative to starting position The maximum and mean shift magnitude taken for all displacement measured over the full treatment session.  The first to last data refers to the first image at the start of treatment followed by the last image set at the completion of treatment.

Patient	Long (mm)	Lat (mm)	Vert (mm)	Vector (mm)
1				
Max	0.70	0.70	0.30	1.03
Mean	0.30	0.30	0.15	0.45
First to Last	-0.70	0.30	0.10	0.77
2				
Max	0.90	0.50	0.40	1.10
Mean	0.36	0.21	0.11	0.43
First to Last	-0.30	-0.30	0.30	0.52
3				
Max	2.40	1.20	1.80	3.23
Mean	0.66	0.51	0.86	1.20
First to Last	-2.40	0.60	-0.50	2.52
4				
Max	0.70	0.70	0.30	1.03
Mean	0.28	0.27	0.10	0.40
First to Last	0.30	-0.50	0.00	0.58
5				
Max	0.90	0.70	0.60	1.29
Mean	0.35	0.30	0.24	0.52
First to Last	-0.60	-0.50	-0.50	0.93

These movements remained within a 1 mm window in any one direction; 0.9 mm in the longitudinal, 0.7 mm in the lateral and 0.6 mm in the vertical directions. The mean error in displacement during the treatment fraction is 0.36 mm in the longitudinal, 0.30 mm in the lateral and 0.24 mm in the vertical direction. Patient 3 had much greater movement and his graph (Figure 4) indicates repeated movement outside of a 1 mm region. Patient 3 has a maximum shift of 2.4 mm in the longitudinal, 1.2 mm in the lateral, and 1.8 mm in the vertical direction at the end of treatment.

We also analyzed the data by looking at the discrete motion from one imaging node to the next; essentially from one beam to the next beam. This allowed us to define the absolute shift between imaging points which would be the effective error in position if patient movement were taking place (Table 3). The maximum movement from one imaging node to the next for patients 1,2,4 and 5 was 0.7 mm in the longitudinal direction, 0.4 mm in the lateral, and 0.4 mm in the vertical direction. For patient 3 the maximum shifts between imaging positions were 2.7 mm in the longitudinal, 2.2 mm in the lateral and 0.7 mm in the vertical direction.

**Table 3:** Patient displacement relative to previous position Shifts are based on progressive imaging sessions. Each value represents the difference between the previous image position and the current position. The mean refers to the average shift from one position to the next

Patient	Long (mm)	Lat (mm)	Vert (mm)	Vector (mm)
1				
Max	0.30	0.40	0.40	0.64
Mean	0.09	0.14	0.15	0.22
2				
Max	0.70	0.40	0.40	0.90
Mean	0.24	0.10	0.12	0.28
3				
Max	2.70	2.20	0.70	3.55
Mean	0.66	0.38	0.18	0.78
4				
Max	0.40	0.40	0.50	0.75
Mean	0.10	0.12	0.11	0.19
5				
Max	0.70	0.40	0.40	0.90
Mean	0.24	0.10	0.12	0.28

We have also calculated the mean movement between imaging nodes for the duration of the treatment. The mean movement between image sequences in the longitudinal direction varied from 0.09 mm to 0.24 mm, from 0.10 mm to 0.14 mm in the lateral direction, and from 0.11 mm to 0.15 mm in the vertical direction (Table 3). The results demonstrate that with continuous imaging and correction the average error in targeting is very small. This data is within CK imaging uncertainty. For patient 3 the value was somewhat larger and was 0.66 mm in the longitudinal direction, 0.38 mm in the lateral and 0.18 mm in the vertical direction. Although the masks are molded to the patient’s cranium, appear stable, and the patient is cautioned not to move, it appears that movement may still take place in certain patient populations. Previous studies have shown that about 1 mm margin for movement is typically sufficient for thermoplastic mask systems [2-4]. However, these studies relied on imaging comparison prior to, and post treatment session, or repeated phantom imaging where no motion would be expected. The current study includes continuous monitoring of movement during treatment. The results of patient 1,2,4 and 5 suggest that typical movement in this small population studied is restricted to a 1 mm region for the duration of treatment. This is similar to data presented by others [2-4,6]. However, the results of patient 3 suggest that a subpopulation of patients may exhibit occasional movement even under well-controlled conditions. The movement is possibly involuntary and may happen during a hiccup, sleep tremor, etc. The clinical significance for this displacement is difficult to understand since the movement appears random and is transient, returning back to a position closer to the initial setup location. In our current system we are imaging and continuously correcting for these movements. Being able to continuously monitor patient position during the full course of SRS would prevent serious displacement within the treatment fraction from being a concern.

## Discussion

We have demonstrated that a thermoplastic immobilization system used for radiosurgery appears to restrict cranial motion within 1 mm in longitudinal, lateral and vertical directions for most patients. This result agrees with previous publications. If the initial setup position is compared to the position at the completion of treatment then a 1 mm margin may appear acceptable in most cases. However, in this study we also monitored continuous movement, at 15-sec intervals, during the duration of the treatments which ranged from 24-64 minutes. During the course of a treatment fraction, we measured movement up to 2.4 mm in the longitudinal direction and 1.8 mm in the vertical direction which returned closer to baseline on subsequent imaging. Others have also shown that thermoplastic systems will sometimes exceed 1 mm linear motion [6] and have cautioned on the use of these immobilization systems without continuous monitoring of patient position [8]. Our results are similar to a recent publication reporting that a subpopulation of cranial SRS patients in thermoplastics masks may move 2 mm to 3 mm [9]. The clinical consequence of these movements is difficult to determine, but clearly in the case of TGN this movement should be reduced to an absolute minimum. In our approach, constant monitoring and position corrections by the CK robot, results in minimal overall error in treatment position. Other systems that do not have a means of frequent positional monitoring may result in poor dose distributions with suboptimal treatment of a target and dose spillage into a region at risk.

## Conclusions

For most patients, our study on non-invasive thermoplastic masks will offer a reliable means of immobilization during radiosurgery. However, a subpopulation of patients may exhibit motion well outside of the typical 1 mm range and may be at risk of suboptimal treatment. Monitoring of patient position, at frequent intervals throughout the treatment fraction, would ensure that this motion is observed allowing intervention when necessary. The intervention can be in the form of continuous correction to the beam path, or beam hold until patient repositioning takes place.
